# Supplementary material for: Regulatory Policies for Alcohol, other Psychoactive Substances and Addictive Behaviours: The Role of Level of Use and Potency. A Systematic Review
Source: Int J Environ Res Public Health. 2019 Oct 4;16(19):3749. doi: 10.3390/ijerph16193749 (PMC6801613; doi:10.3390/ijerph16193749)

Supplementary Materials

Table S1: Search strategy

No.	Searches	Results
**Study type terms**		
1	(systematic review or meta-analysis).mp. [mp=ti, ab, ot, nm, hw, fx, kf, ox, px, rx, ui, sy, tn, dm, mf, dv, kw, dq]	664414
2	limit 1 to humans	588466
**Exposure terms**		
3	(marijuana or cannabis).mp. or exp Cannabis/ or exp "cannabis use"/ or exp cannabis smoking/ or exp "Cannabis (genus)"/	82169
4	opioids.mp. or exp Analgesics, Opioid/ or exp opiate/	473840
5	exp tobacco smoke/ or exp "tobacco use"/ or exp tobacco/ or exp smokeless tobacco/ or exp chewing tobacco/ or tobacco.mp. or exp tobacco consumption/	587217
6	exp gambling/ or gambling.mp. or exp pathological gambling/	18591
7	(alcohol* adj3 (drink* or consum* or intake)).mp. or exp drinking behavior/ or exp alcoholic beverage/	333308
8	3 or 4 or 5 or 6 or 7	1383650
**Different policy and regulation terms**		
9	exp Policy/ or exp Public Policy/ or policy.mp. or exp Health Policy/ or regulation.mp.	3543740
10	exp Policy/ or exp Public Policy/ or policy.mp. or exp Health Policy/ or regulation.mp. or exp regulatory mechanism/	4009570
11	exp Policy/ or exp Public Policy/ or policy.mp. or exp Health Policy/ or regulation.mp. or exp regulatory mechanism/ or exp Policy Making/	5032816
**Potency and dosage terms**		
12	exp drug dose/ or exp maximum permissible dose/ or exp dose/ or exp dose calculation/ or dose.mp. or exp dose response/ or exp maximum tolerated dose/ or exp recommended drug dose/ or potency.mp.	3956167
13	2 and 8 and 9 and 12	173
14	remove duplicates from 13	167
15	2 and 8 and 10 and 12	281
16	remove duplicates from 15	275
17	2 and 8 and 11 and 12	484
18	remove duplicates from 17	474

Databases searched: OVID Medline and Embase, August 19th 2019

Figure S1: PRISMA flow-chart on selection of relevant reviews

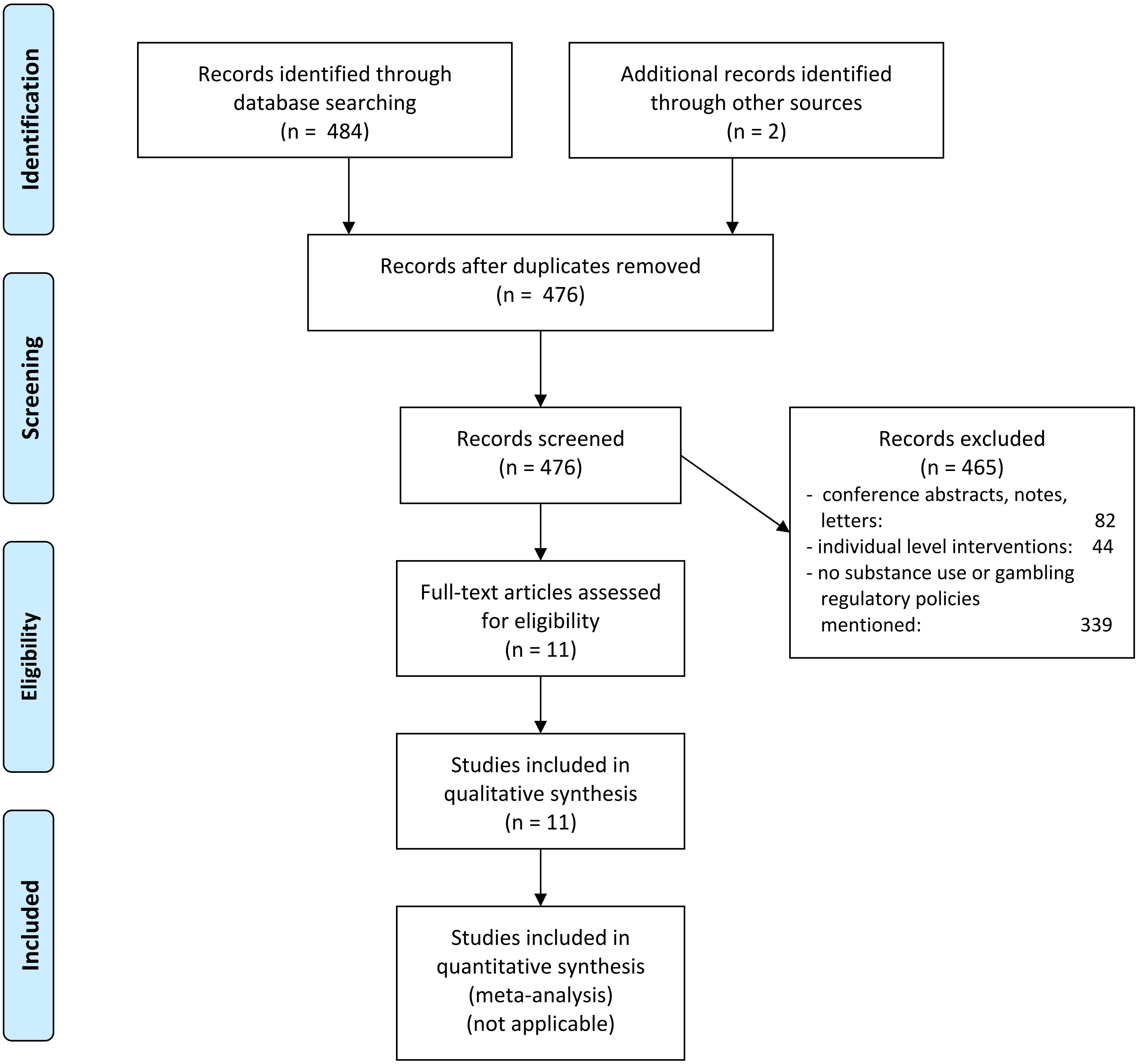

Supplement: Supplementary file 1 [file ijerph-16-03749-s001.pdf]
